# Diagnostic Accuracy of Clinical Tests Assessing Ligamentous Injury of the Talocrural and Subtalar Joints: A Systematic Review With Meta-Analysis

**DOI:** 10.1177/19417381211029953

**Published:** 2021-07-21

**Authors:** Fredh Netterström-Wedin, Mark Matthews, Chris Bleakley

**Affiliations:** † Department of Community Medicine and Rehabilitation, Umeå University, Umeå, Sweden; ‡ Sport and Exercise Science Research Institute, Ulster University, Belfast, UK; § School of Health Sciences, Faculty of Life and Health Sciences, Ulster University, Jordanstown Campus, Antrim, UK

**Keywords:** diagnosis, ankle, examination, ligament, meta-analysis

## Abstract

**Context:**

Ankle sprains are the most common acute musculoskeletal injury. Clinical tests represent the first opportunity to assess the sprain’s severity, but no systematic review has compared these tests to contemporary reference standards.

**Objective:**

To determine the diagnostic accuracy of clinical tests assessing the talocrural and subtalar joint ligaments after ankle sprain.

**Data Sources:**

CINAHL, EMBASE, MEDLINE, hand-searching, and PubMed-related article searches (inception to November 18, 2020).

**Study Selection:**

Eligible diagnostic studies compared clinical examination (palpation, joint laxity) against imaging or surgery. Studies at a high risk of bias or with high concerns regarding applicability on Quality Assessment of Diagnostic Accuracy Studies-2 were excluded from the meta-analysis.

**Study Design:**

Systematic review and meta-analysis.

**Level of Evidence:**

Level 3a.

**Data Extraction:**

True-positive, false-negative, false-positive, and true-negative findings were extracted to calculate sensitivity, specificity, and likelihood ratios. If ordinal data were reported, these were extracted to calculate Cohen’s kappa.

**Results:**

A total of 14 studies met the inclusion criteria (6302 observations; 9 clinical tests). No test had both sensitivity and specificity exceeding 90%. Palpation of the anterior talofibular ligament is highly sensitive (sensitivity 95%-100%; specificity 0%-32%; min-max; n = 6) but less so for the calcaneofibular ligament (sensitivity 49%-100%; specificity 26%-79%; min-max; n = 6). Pooled data from 6 studies (885 observations) found a low sensitivity (54%; 95% CI 35%-71%) but high specificity (87%; 95% CI 63%-96%) for the anterior drawer test.

**Conclusion:**

The anterior talofibular ligament is best assessed using a cluster of palpation (rule out), and anterior drawer testing (rule in). The talar tilt test can rule in injury to the calcaneofibular ligament, but a sensitive clinical test for the ligament is lacking. It is unclear if ligamentous injury grading can be done beyond the binary (injured vs uninjured), and clinical tests of the subtalar joint ligaments are not well researched. The generalizability of our findings is limited by insufficient reporting on blinding and poor study quality.

**Registration:**

Prospero ID: CRD42020187848.

**Data Availability:**

Data are available in a public, open access repository on publication, including our RevMan file and the CSV file used for meta-analysis: http://doi.org/10.5281/zenodo.4917138

Each year, over 300,000 people present to UK emergency departments with ankle sprain (~800 per day).^5^ Many occur during sporting or recreational activity because of excessive inversion and internal rotation of the ankle at high velocity.^27^ Ankle sprains are often regarded as innocuous injuries, but up to 70% of the patients develop chronic ankle instability; characterized by mechanical laxity, subjective feelings of giving way, persistent pain and reinjury.^27^ In the United Kingdom, the total average cost associated with a lateral ankle sprain is estimated at £940.^10^ The high incidence of chronic symptoms, risk of recurrence, and long-term risk of developing posttraumatic osteoarthritis further contribute to the significant socioeconomic burden of lateral ankle sprains.^27^

Limited data inform the causality of chronic ankle instability.^4^ An emerging hypothesis is that poor prognosis after ankle sprain is mediated by inadequate clinical examination. The primary concerns are that existing clinical tests often fail to identify microinstabilities of the ankle joint complex; which consists of the anterior talofibular ligament (ATFL), calcaneofibular ligament (CFL), and the posterior talofibular ligament (PTFL).^22^ Also, few tests target the primary stabilizers of the subtalar joint, consisting of the interosseous talocalcaneal ligament (ITCL), cervical ligament (CL), and the anterior capsular ligament (ACL). Recommendations for clinical examination of suspected lateral ligamentous injury continue to be underpinned by palpation and manual stress tests (eg, anterior drawer and talar tilt).^13^ However, only 2 reviews^54,55^ have systematically reported their diagnostic accuracy. The most recent review^54^ included just 5 studies, with the majority limited to arthrographic (stress radiography) reference standards.

We must reexamine the diagnostic utility of clinical examination techniques in this field by also including contemporary reference standards (ultrasound, magnetic resonance imaging [MRI], and arthroscopy).^7^ Diagnostic accuracy may be optimized through test clustering, and through the inclusion of new index tests (such as modified drawer tests), but this has not been systematically examined. A key part of clinical examination should be to differentiate isolated versus combined injuries of the talocrural and subtalar joints, and use this to determine prognosis, or guide management decisions. MRI and arthroscopy can consistently identify concomitant damage to primary stabilisers of the subtalar joint, but it is unclear if clinical tests have comparable diagnostic utility.

## Methods

### Protocol and Registration

We used the Preferred Reporting Items for Systematic Reviews and Meta-Analysis of Diagnostic Test Accuracy Studies (PRISMA-DTA)^45^ for our review.

We prospectively drafted our study protocol to PROSPERO on May 20 2020, registration ID: CRD42020187848.

### Eligibility Criteria

We assessed original research for eligibility using the criteria presented in Table 1, with no restrictions on the language of the article nor the publication year. Most criteria were decided on a priori, as part of the PROSPERO protocol. However, arthroscopy as an inclusion criterion was extended to include other surgical techniques as well, and avulsion fractures as an exclusion criterion were omitted to broaden the eligibility criteria.

**Table 1.** PICOTS criteria for inclusion and exclusion of studies

Parameters	Inclusion Criteria	Exclusion Criteria
**P**opulation	Ankle sprain	Fractures
**I**ndex test	Any clinical test aiming to reproduce symptoms or assess joint stability	Surgical or imagery stress tests, testing delivered under anaesthesia
**C**omparator	Arthrogram, arthroscopy, magnetic resonance imaging, stress radiography, surgery, or ultrasound	
**O**utcome measure	Ascertain the presence or absence of ligamentous ankle injury	Studies with insufficient information to compute a 2 × 2 contingency table to calculate sensitivity and specificity
**T**ype of study	Prospective cohort, diagnostic case-control studies or retrospective studies	Cadaveric studies, case series, systematic reviews
**S**etting	Any setting	

### Search

**Figure 1:**
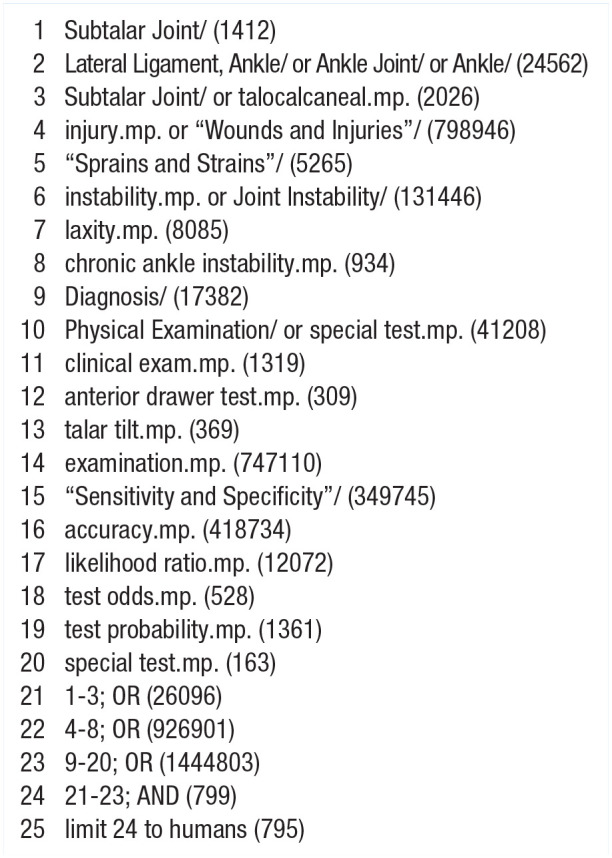
MEDLINE search terms (number of hits).

We conducted electronic database searching of EBSCOhost and Ovid: searching CINAHL, EMBASE, and MEDLINE from inception to November 18, 2020. We used the same search terms for all three databases. We also performed PubMed-related article searches for all studies meeting inclusion criteria from the previous database searches. Finally, we examined the references of our included studies and previous systematic reviews. Our search strategy and the number of hits for MEDLINE can be seen in Figure 1.

### Study Selection

Two reviewers independently screened the title and abstract of every identified record. Afterward, both reviewers presented their respective articles and examined the full-text versions separately. If full-text articles contained insufficient information to decide eligibility, we contacted the corresponding authors for additional details. Disagreements regarding final inclusion were fully resolved through consensus without the need for a third reviewer. After inclusion criteria had been met for our systematic review, we also considered each article for meta-analysis. We excluded retrospective and case-control studies from the meta-analysis because of the risk of these study designs to overestimate diagnostic accuracy. We also excluded studies at a high risk of bias or with high concerns regarding applicability from the meta-analysis.

### Risk of Bias in Individual Studies

Two reviewers performed an independent methodological assessment of the included studies, using the Quality Assessment for Diagnostic Accuracy Studies-2 (QUADAS-2)^68^ tool. There are 4 domains to QUADAS-2: (1) Patient selection: Ideally, all eligible patients should be consecutively enrolled and have a suspected injury relevant to the research question. Convenience sampling, case-control designs, and inappropriate exclusions risk introducing bias in the form of overestimated measures of diagnostic accuracy, as the patient spectrum is not representative of clinical practice. (2) Index test: To minimize the risk of bias, index testing should be interpreted without knowledge of reference test results. Also, the conduct of the index test should be sufficiently described to permit replication, as deviations in execution could affect the generalizability of the findings. (3) Reference standard: Since estimates of diagnostic test accuracy are based on the presumption that the discriminatory properties of the reference standard are perfect, the sensitivity and specificity of the reference standard must be sufficient to correctly diagnose the presence or absence of the injury in question. The reference standard should also be interpreted without prior knowledge of the index test. (4) Flow and timing: Both the index test and the reference standard should be delivered as close in time to each other as possible. A prolonged time-span introduces confounding effects from intermediate interventions or regression to the mean, thus leading to non-valid study findings.^53,62^ After we had performed independent quality assessments, a consensus meeting was organized, during which we reached full agreement.

### Data Items

Information regarding study setting (eg, private, public, sports, primary care, emergency department); study design (prospective, retrospective, case-control); population demographics (age, gender, level of sporting participation, time since injury); details of index tests and reference standards (testing protocol, the definition of a positive test outcome, flow, and timing) were extracted independently and in duplicate into a predefined form by 2 reviewers. The extracted information was then reviewed and confirmed by a third reviewer, who compared the completed forms to each other and the original research reports.

### Synthesis of Results

We produced 2 × 2 contingency tables based on the true-positive, false-positive, true-negative, and false-negative findings of the included studies. With this information, we used Review Manager 5.4 software^9^ to compute sensitivity and specificity values and their respective 95% confidence intervals (CIs). Sensitivity values are representative of the proportion of those with injury correctly classified as injured, while specificity values are representative of the proportion of those without injury correctly classified as healthy.

If ordinal-level data were reported, these were extracted and analysed to see if clinical tests can accurately grade the degree of injury. We calculated the interrater agreement between index test and reference test with weighted Cohen’s kappa (linear weighting), using an online calculator.^25^ According to McHugh,^44^ kappa values for agreement are to be interpreted as follows: 0 to 20 = none; 21 to 39 = minimal; 40 to 59 = weak; 60 to 79 = moderate; 80 to 90 = strong; >90 = almost perfect.

All data extraction into Review Manager 5.4 was done independently and in duplicate by 2 reviewers. A third reviewer verified the extracted data by comparing the results between the 2 reviewers and by cross-referencing against the original research reports. If discrepancies were noticed between the 2 reviewers responsible for data extraction, the third reviewer decided what data to present. The primary author then performed all statistical analyses.

### Meta-Analysis

We performed HSROC (hierarchical summary receiver operating characteristic) and bivariate meta-analyses with MetaDTA 2.0 software.^17,48^ We calculated pooled summary estimates of test sensitivity, specificity, and positive and negative likelihood ratios (LRs), each with 95% CI. LRs are considered a useful diagnostic metric and represent the prevalence of positive tests in those with injury versus those without (LR+) and the prevalence of negative tests in those that are healthy versus those that are not (LR−).^12^ We plotted the pooled LRs in Fagan’s nomogram,^16^ to examine the change in pre- to posttest probability after positive and negative tests. We estimated the pretest probability through the median disease prevalence of studies eligible for meta-analysis. To determine heterogeneity, we used the Cochran *Q* test (*P* < 0.05 indicating presence of heterogeneity) and the *I*^2^ statistic. *I*^2^ values of 0% to 40%, 30% to 60%, 50% to 90%, and 75% to 100% were considered nonimportant, moderate, substantial, and significant levels of heterogeneity, respectively.^29^ This univariate analysis of heterogeneity was done with OpenMetaAnalyst software.^66^ We also considered the correlation between sensitivity and specificity during bivariate modeling, the distance between each study and the HSROC curve, and the width of the prediction ellipse. Since some amount of heterogeneity is to be expected in studies on diagnostic test accuracy, we used random-effects modeling for all analyses.^43^

### Additional Analyses

We had prespecified subgroup analyses planned as part of our PROSPERO protocol, using the clinician’s experience and the time since injury as covariates. However, because of the low number of studies meeting methodological criteria for meta-analysis, we deemed this inappropriate.

#### Counting Inconclusive Findings

According to Simel et al,^57^ inconclusive findings can either be termed “uninterpretable,” “intermediate,” or “indeterminate.” Uninterpretable results are when the patient, for whatever reason, cannot adequately undergo the intended test. Intermediate test results raise the disease’s probability above what is deemed “healthy,” but not enough to be considered “diseased.” Indeterminate results add no additional value to the original probability of disease. It is often prudent to include inconclusive findings in the primary analysis to not risk overestimating the test’s diagnostic accuracy.^56^ For both the primary analysis and the meta-analysis, we grouped “uninterpretable” test results as injury positive, and “intermediate” test results as injury negative. The uninterpretable results were because of either excessive pain or swelling.^49,50,63,65^ We believe that counting these patients as injury positive reflects what would have been done in the clinical setting, since clinicians would intuitively raise their suspicion of ligamentous damage if the patient presented with excessive levels of the aforementioned clinical signs. We grouped intermediate findings^49,63^ (ie, tests were the clinician could not decide whether the patient had enough laxity to be determined injured vs uninjured) as disease negative, since the positivity criteria for stress testing is the definitive presence of increased joint laxity. We encountered no “indeterminate” tests results in the included studies. Appendix 1 (available in the online version of this article) contains the inconclusive index test findings and the diagnostic yield as a percentage of manual stress tests used for diagnosis versus the number of patients intended to diagnose.

### Patient and Public Involvement

Patients were not involved in the development of the research question or its outcome measures, the conduct of the research, or preparation of the manuscript. Dissemination of results to these groups is not applicable.

## Results

### Study Selection

**Figure 2.**
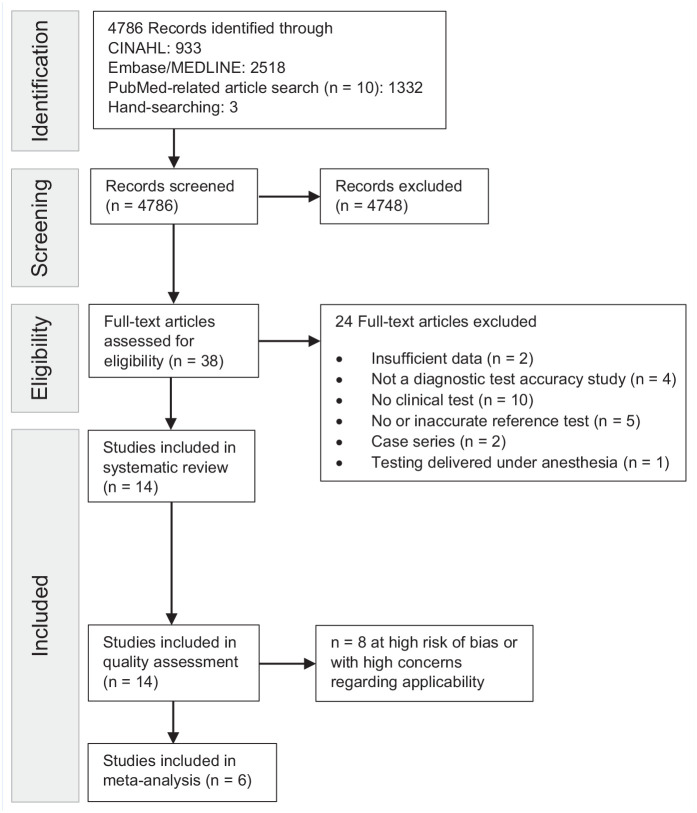
Study flow diagram. Two authors independently examined each record for study inclusion eligibility and suitability for the subsequent meta-analysis.

Our search yielded 4786 records. After the initial title and abstract screening, we assessed 38 full-text articles for final eligibility. We excluded 24 articles because of the following reasons: insufficient data^18,34,58^ (n = 2), not a diagnostic test accuracy study^1,30,37,46^ (n = 4), no clinical test^2,3,20,24,31-33,36,41,52^ (n = 10), no or inaccurate reference test^15,28,42,47,51^ (n = 5), case series^6,60^ (n = 2), and testing delivered under anesthesia^69^ (n = 1). We contacted 3 authors to help clarify details related to their data,^14,23,58^ with none responding. In total, 14 articles met the inclusion criteria of our systematic review, with 6 of them contributing to meta-analysis. Figure 2 contains a flowchart of the study selection process.

### Study Characteristics and Results

Appendix 2 (available online) provides detailed information on study characteristics. Two studies were retrospective reviews,^8,26^ the rest being diagnostic case-control,^23^ clinical trials,^63^ or prospective cohort studies (n = 10).^11,14,19,21,38,40,49,50,64,65^ Studies included an aggregate of 2391 participants. The proportion of women within each study ranged from 23% to 51%. Seven studies were conducted in emergency departments^63-65,19,40,49,50^ and 7 in outpatient clinics.^8,11,14,20,23,26,38^ Eleven of 14 studies included sporting populations.^11,19,21,23,26,38,40,49,50,63,64^ Only Gremeaux et al^26^ and van der Ent^64^ specified the level of play, the majority of which were recreational practitioners (85%) and amateur competitors (46%), respectively. Most studies included participants with recent (≤7 days) ankle injuries,^14,19,26,40,49,50,63-65^ with the remainder enrolling participants with either chronic ankle instability,^8,23,38^ or a mixture of both.^11^ In addition to the binary classification of injury status, 2 of the 14 studies also assessed the level of agreement for ordinal injury grading between index and reference testing.^8,21^

The reference standards used were arthrography^19,49,50,63-65^ (n = 6), arthroscopy or surgery^8,41^ (n = 2), MRI^14,23^ (n = 2), and ultrasound^11,21,26,38^ (n = 4). Two of the 6 studies using arthrography as the reference standard did not aim to differentiate between the affected ligaments during reference testing, counting any ligament sprain as a positive finding.^19,65^ One study^63^ provided detailed information for arthrography criteria, but insufficient information in cross-reference to the index test results to differentiate between what ligaments were involved beyond the ATFL. Two of 4 ultrasonographic studies defined a positive reference test as a partial to complete ATFL rupture.^11,38^ Croy et al^11^ was the only study that numerically quantified the degree of laxity during the ultrasound examination, and defined a positive finding as anterior talar displacement of ≥3.7 mm, which constituted twice the standard deviation of the values from the healthy control group. George et al^21^ and Gremeaux et al,^26^ also using ultrasound as the reference standard, differentiated between ATFL and CFL tearing. De Simoni et al^14^ also differentiated between injury of the 2 ligaments, but via MRI. Gomes et al^23^ was the only study that did not disclose any details on what defined a positive finding during reference testing.

Five studies explicitly stated that they received financial aids through noncommercial research grants.^11,19,38,40,63^ One study^23^ noted that no grants whatsoever were received, and another 2 made clear that no commercial grants that would put the authors at a conflict of interest were received.^21,65^ Six studies did not state any details on funding.^8,14,26,49,50,64^

Appendix 3 (available online) has details of index test execution and positive test interpretation. The index test most commonly studied was the anterior drawer test^8,11,19,21,23,38,49,50,63,65^ (n = 10) followed by palpation of the ATFL and the CFL (both n = 6).^14,19,26,40,64,65^ Other stress tests used were the reverse anterior drawer^38,40^ (n = 2), the anterolateral drawer^38^ (n = 1), heel adduction^19^ (n = 1), talar tilt^19,21,49,63^ (n = 4), and supination test^19,40^ (n = 2). The anterior drawer test was performed at varying degrees of plantar flexion, ranging from neutral^11,50^ to 60°.^49,63^ Most studies described a knee flexed test position,^8,11,19,21,23,38,65^ either lying supine or seated. Positive test interpretation differed and was based on either increased laxity^8,11,19,21,23,38,49,50,63^ or the presence of a dimple sign.^65^ One study^40^ stated that they had applied an anterior drawer test and a talar tilt test; however, the test description and images seem to align more with the reverse anterolateral drawer test^38^ and the supination test.^19^

Details on test execution were scarce for studies examining palpation: Most studies failed to report the exact point for palpation across the ligaments, and the amount of force applied. Only 1 study^15^ stated that the entirety of the ligament was palpated for the pain punctum maximum and another^65^ study stated that the ATFL was palpated both by the tip of the fibula and over the talus.

### Risk of Bias Within Studies

Table 2 summarizes our QUADAS-2 assessment. Three studies—Croy et al,^11^ George et al,^21^ and Li et al^38^—completed all QUADAS-2 domains with a low risk of bias and with low concerns regarding applicability. Most studies had a low risk of bias regarding patient selection and index testing. Only Gomes et al,^23^ using a case-control design, did not disclose patient enrollment and exclusion criteria.

**Table 2.** Quality Assessment of Diagnostic Accuracy Studies-2 (QUADAS-2) summary of findings

	Risk of Bias				Applicability Concerns		
Authors and Year	Patient Selection	Index Test	Reference Standard	Flow and Timing	Patient Selection	Index Test	Reference Standard
Cho et al 2016	?	☺	?	☺	☹	☺	☺
Croy et al 2013	☺	☺	☺	☺	☺	☺	☺
De Simoni et al 1996	☺	☺	☹	☹	☺	☺	☺
Funder et al 1982	☺	☺	?	☹	☺	☺	☺
George et al 2020	☺	☺	☺	☺	☺	☺	☺
Gomes et al 2017	☹	☺	?	☹	☺	☺	☺
Gremeaux et al 2009	?	?	?	☺	☺	☺	☺
Li et al 2020	☺	☺	☺	☺	☺	☺	☺
Lindstrand 1976	☺	☺	?	☺	☺	☺	☺
Prins 1978	☺	?	☺	☺	☺	☺	☺
Raatikainen et al 1992	?	☺	?	☺	☺	☺	☺
van den Hoogenband et al 1984	☹	☺	☺	☺	☹	☺	☺
van der Ent 1984	☺	☺	?	☹	☺	☺	☺
van Dijk et al 1996	☺	?	☺	☺	☺	☺	☺

☺low risk; ☹high risk; ? unclear risk

There was an unclear risk of bias for test interpretation in 9 of the included studies. Prins^49^ performed reference testing before index testing, and Gremeaux et al^26^ provided insufficient details to determine test order. Van Dijk et al^65^ mentioned that a positive anterior drawer test was sometimes unwittingly interpreted based on pain response instead of increased laxity. Still, it is unclear how many patients were deemed injured based on the unintended pain criteria. In a further 7 studies, it was unclear if the reference test was interpreted without knowledge of the results of the previous index tests.^8,19,23,26,40,50,64^

For study flow and timing, 4 studies carried a high risk of bias.^14,19,23,64^ De Simoni et al^14^ employed an inappropriate time interval between index testing and reference testing (mean delay 9.4 days). As the included patients were examined acutely (0-19 days after injury), each day of delay represents a relatively larger proportional discrepancy in study flow and timing, when compared with more prolonged periods of injury. Funder et al^19^ and van der Ent^64^ limited their reference standard examination to patients with high clinical suspicion and positive index tests, resulting in verification bias. Van der Ent’s^64^ cohort was further stratified based on the arthrographic findings for the subsequent treatment intervention. However, in the strata serving as the control group, insufficient information regarding the affected structures made it impossible to discern the diagnostic accuracy of the different palpation tests for this subset of patients. The control group in Gomes et al^23^ did not receive the reference standard, and it is unclear whether or not their data were used to calculate the sensitivity and specificity values of the studied clinical tests.

### Results of Individual Studies

**Figure 3.**
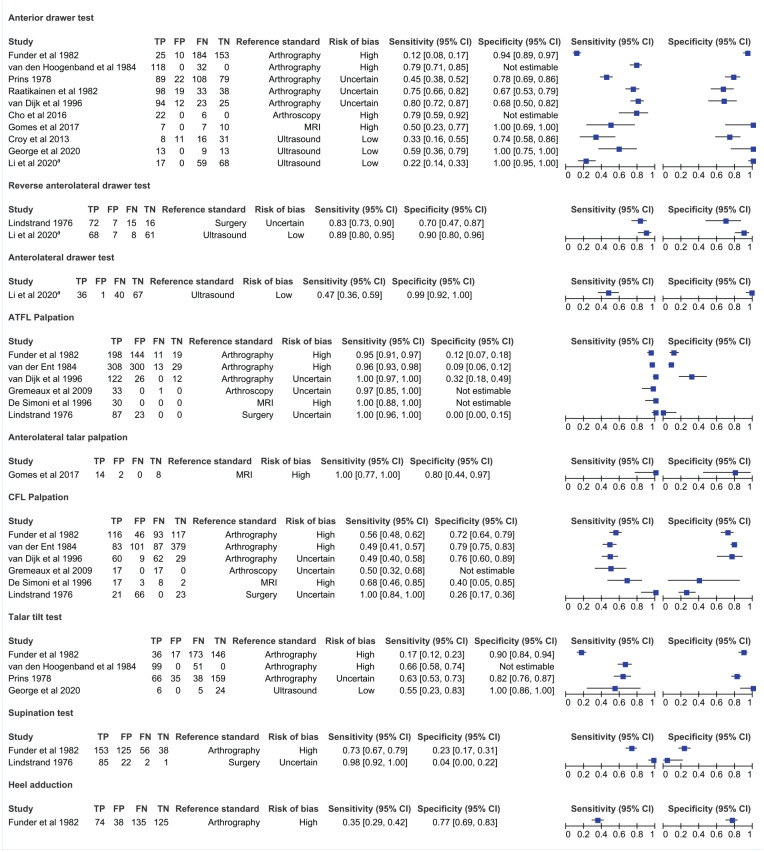
Individual diagnostic test accuracy study results for the 9 clinical tests identified. FN, false negative; FP, false positive; MRI, magnetic resonance imaging; TN, true negative; TP, true positive. aSeventy-seven patients were examined by 2 examiners.

Figure 3 presents the diagnostic accuracy of each test from the individual studies. In total, 6302 observations from 14 studies spread over 9 clinical tests contributed to the narrative synthesis.

#### Manual Stress Tests

The drawer test has higher specificity than sensitivity for diagnosing injury to the ATFL,^8,21,23,38,40,49,50,63^ any lateral ligamentous injury,^19,65^ or excessive joint instability.^11^ This was typically observed, regardless of the technique employed: anterior drawer test^8,11,19,21,23,38,49,50,63,65^ (sensitivity range 12%-80%, specificity range 67%-100%); anterolateral drawer test^38^ (47% sensitivity and 99% specificity); reverse anterolateral drawer test^38,40^ (sensitivity range 83%-89%, specificity range 70%-90%). The talar tilt test^19,21,49^ and the heel adduction test^19^ were also more specific than sensitive for diagnosing any lateral ligamentous injury^19,63^ or injury to the CFL^21,49^ displaying 17% to 66% sensitivity with 82% to 100% specificity, and 35% sensitivity with 77% specificity, respectively. Conversely, the supination test^19,40^ proved more sensitive (73%-98%) than specific (4%-23%) for diagnosing ATFL injury^40^ or any lateral ligamentous injury.^19^

#### Palpation

Palpation is more sensitive than specific. Anterolateral talar palpation^23^ displayed a perfect sensitivity (100%) and 80% specificity for diagnosing injury to the ATFL. Direct palpation of the ATFL^14,19,26,40,65^ consistently showed high sensitivity (95%-100%) across 6 studies but low (0%-32%) specificity when diagnosing ATFL rupture^14,26,40,64^ or any affected lateral collateral ligament.^19,65^ Palpation of the CFL^14,19,26,64,65^ had worse sensitivity, ranging between 49% and 100%, while specificity ranged between 26% and 79% for diagnosing partial to total tearing of the CFL^14,26,40,64^ or any lateral ligamentous tear.^19,65^

No diagnostic test accuracy study examining clinical tests for the subtalar joint met our inclusion criteria.

### Meta-Analysis

**Figure 4.**
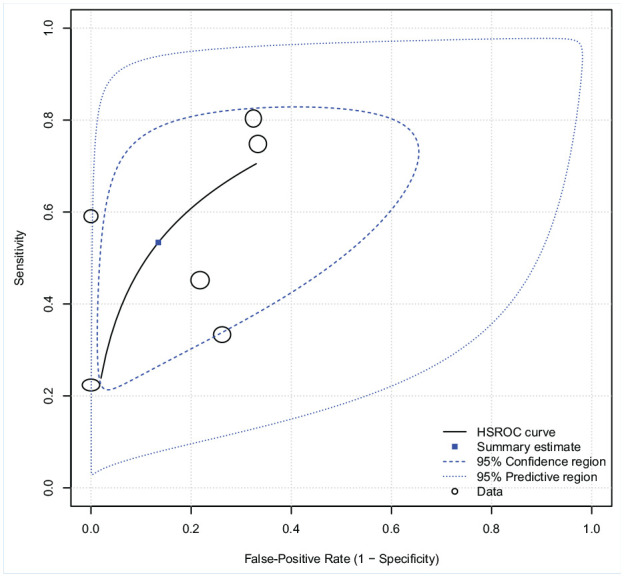
Hierarchical summary receiver operating characteristic curve (HSROC) (and summary point) of the anterior drawer test’s pooled sensitivity and specificity. The distance between the study points and the summary curve, as well as the width of the prediction ellipse, hints toward differences in positivity threshold (ie, the amount of laxity necessary for the clinician to classify the patient as injured) for the included studies.

Six studies (885 observations) examining the anterior drawer test were included in our meta-analysis.^11,21,38,49,50,65^ Using a bivariate model, the pooled metrics for the anterior drawer test were: sensitivity 54% (95% CI 35%-71%), specificity 87% (95% CI 63%-96%), LR+ 3.97 (95% CI 1.50-10.47), and LR− 0.54 (95% CI 0.39-0.75) (n = 6). Sensitivity and specificity were negatively correlated (−0.73). When modeled independently, sensitivity displayed significant heterogeneity (*I*^2^ = 94.2%, Cochran’s Q*P* < 0.001) and specificity displayed substantial heterogeneity (*I*^2^ = 62.1%, Cochran’s Q *P* = 0.022). It is plausible that a threshold effect in test interpretation (ie, the amount of laxity required during translation for the clinician to say that the patient is injured) explains some of the between-study variations in sensitivity and specificity.^61^ A threshold effect is further supported by the distance of the studies from the summary curve and the prediction ellipse (Figure 4).^43^

**Figure 5.**
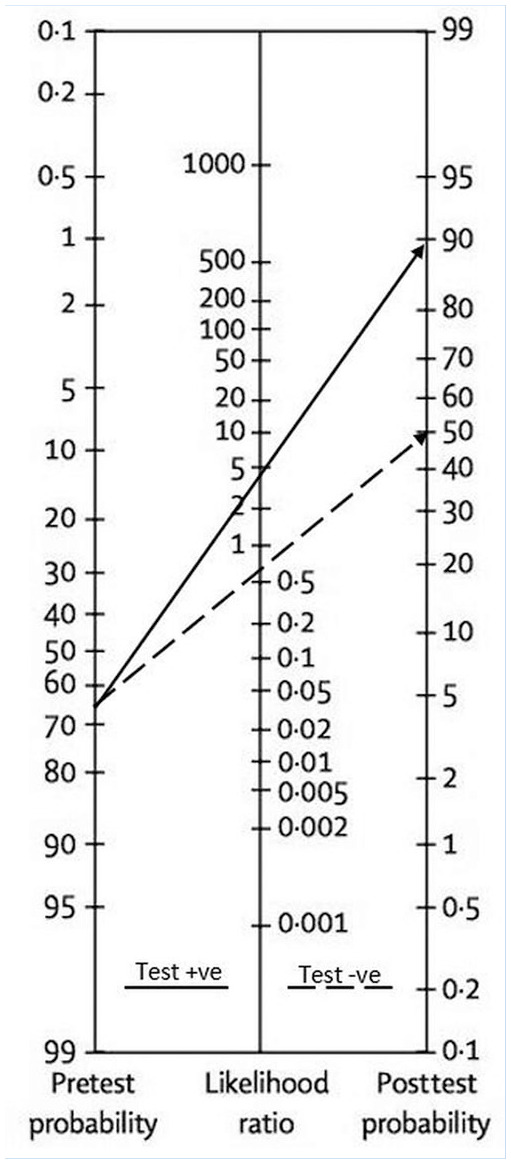
The pooled likelihood ratios of the anterior drawer test incorporated into Fagan’s nomogram. The median disease prevalence of studies undergoing meta-analysis was used as the pretest probability of injury (any lateral ligamentous injury). A positive anterior drawer test is associated with a much greater shift in posttest probability of ligamentous damage in comparison to a negative test result.

The median prevalence for any lateral ankle ligament injury was 65% (36%-76% min-max) in the studies underdoing meta-analysis. Using this percentage as the pretest probability of injury for Fagan’s nomogram, a positive anterior drawer test (LR+ 3.97) increases the clinical likelihood of lateral ligamentous injury to 88%. A negative test result (LR− 0.54) is associated with a smaller drop in probability to 50% (Figure 5).

### Assessing the Degree of Ligamentous Injury

Cho et al^8^ investigated the discriminatory capabilities of the anterior drawer test in comparison to arthroscopic grading of perceived joint laxity on a 3-point ordinal scale (subtle/moderate/severe laxity; grade 1/2/3). Although 77% agreement was observed between the clinical grading and arthroscopic grading, this was no greater than chance agreement ([index test: 0, 6, 20] [reference test: 0, 0, 26] [κ = 0, weighted Cohen’s kappa]), implicating limited use of the clinical test in differentiating between moderate and severe cases of joint laxity.

George et al^21^ used a similar clinical grading scale (no/some/gross laxity; grade 1/2/3) and cross-referenced the findings with stress ultrasound examination (intact/partially torn/completely torn ATFL ligament; grade 1/2/3). However, George et al^21^ included a larger sample and patients of varying injury severity. In this study, the grading of perceived laxity during anterior drawer testing and the amount of ATFL tearing found during stress ultrasound examination reached moderate agreement ([index test: 10, 12, 13] [reference test: 8, 5, 22] [κ = 0.53, weighted Cohen’s kappa]).

George et al^21^ also examined the agreement between clinical grading during the talar tilt test and the degree of CFL rupture during dynamic ultrasonography. The proportion of unaffected ankles were greater (15 vs 8) for the CFL in comparison to the ATFL, and tears were evenly distributed between partial (n = 5), and total (n = 5) ruptures. Still, the interrater agreement between clinical and ultrasound grading of CFL status was almost identical to that of the anterior drawer test and ultrasound ATFL grading, displaying moderate agreement ([index test: 16, 14, 5] [reference test: 15, 10, 10] [κ = 0.52, weighted Cohen’s kappa]).

## Discussion

### Principal Findings

Lateral ankle sprains are the most common acute musculoskeletal injury. They can result in damage to any of the primary lateral ligaments spanning the talocrural (ATFL, CFL, PTFL) and subtalar joints (ITCL, CL, ACL). Diagnosis and prognosis postsprain should be informed by the number of ligaments damaged and the severity of the tear. This review suggests accurate clinical diagnosis is limited to 1 ligament in the ankle complex; the ATFL. Diagnosis of injury to the ATFL achieves maximum accuracy through clustering of ligament palpation (highly sensitive) and anterior drawer testing (highly specific). The talar tilt test can help rule in injury to the CFL, but sensitive tests aimed at the ligament are lacking. There is limited and conflicting evidence that clinical tests can provide an accurate assessment of injury severity. Studies examining the diagnostic accuracy of clinical tests aimed at the subtalar ligaments are lacking.

### Explanations and Implications for Clinicians

Ligamentous injury to the ankle typically follows a hierarchical pattern. The ATFL is the weakest lateral ligament and is involved in ~80% of ankle sprains.^40^ The evidence suggests that clinical assessment of the ATFL necessitates a combination of palpation and anterior drawer testing to differentiate between injured and uninjured patients accurately. Although palpation techniques were poorly described, we would suggest that the entire ligament is examined, with tenderness at any point indicating a positive finding. The accuracy of the anterior drawer test may be moderated by the test setup, the positivity threshold, and the timing of the test. Traditionally, this test involves moving the heel anteriorly on the tibia. High accuracy was also achieved using a reverse drawer technique,^38,40^ whereby the tibia was pushed posteriorly on a fixed heel. A common feature of both methods was that patients were positioned in knee flexion and plantarflexion. Biomechanical studies corroborate these joint positions, ensuring minimal tension at the triceps surae and maximal recruitment of the ATFL.^33,35^

The positive predictive value of the anterior drawer test may be enhanced further by adopting a high threshold for positivity. This includes interpreting subtle laxities^11,21^ and intermediate results^49,63^ as negative. Three studies^49,64,65^ validate the notion that the accuracy of clinical examination is maximized when undertaken in a delayed (2-7 days) versus acute (<48 hours) setting. The CFL is the only ligament in the lateral collateral complex that crosses both the talocrural and subtalar joints,^22^ and therefore plays an essential role in the lateral stability of the ankle.^67^ Given that peroneal tendons and sheaths cover the majority of the CFL,^22^ it is unsurprising that palpating the ligament provides limited diagnostic value. Although we found consistent evidence that the talar tilt test has excellent specificity, and is useful for ruling in injury to the CFL,^19,21,49^ caution is required when interpreting a negative test. This finding supports the hypothesis that some instabilities of the lateral ligament complex are occult to clinical examination, which may mediate the risk of inadequate management and development of chronic ankle instability.^4^ A related limitation is that we cannot present any clinical tests that are suitable for diagnosing injury to the subtalar ligaments (ITCL, CL, ACL). This is a critical gap in the current evidence base, as differentiating between an isolated versus combined injury of the talocrural and subtalar joints is fundamental for accurate prognostication and clinical management decisions.

### Strength and Limitations

Our study is the first meta-analysis examining the accuracy of clinical testing commonly used for diagnosing ankle sprains. Other studies have reviewed the evidence in this field,^54,55^ but trial numbers were limited (n = 5), with the majority limited to radiographic reference standards. The current review includes data from 6302 observations across 14 trials, including higher quality, contemporary reference standards (ultrasound, MRI, and arthroscopy). Although only 2 studies incorporated the current gold standard reference (arthroscopy or surgery), a previous meta-analysis showed that high diagnostic accuracy is possible using MRI, ultrasound, or stress radiography (81%-99% sensitivity and 79%-91% specificity).^7^ Still, as these reference standards are not perfect (and showcase variability), the diagnostic accuracy of the clinical tests of many of our included studies should be interpreted accordingly. Only 3 of the 14 studies that we included had a low risk of bias across all QUADAS-2 domains. Verification bias was the most frequent, because of either improper time frames between the index and reference test or selective criteria. The generalization of our findings is also affected by poor reporting of test interpretation: Being commonly ambiguous and presenting with an unclear risk of bias. Only 1 study made direct comparisons between modified techniques for routine stress tests,^38^ and just 2 studies incorporated an ordinal scale to grade injury severity.^8,21^ As their results were contradictory, it is unclear if clinical tests of the talocrural joint can grade ligament damage beyond the binary. This review focuses on lateral ligament injuries, but we acknowledge that ankle sprains can also involve the ankle syndesmosis. Injuries to the syndesmosis will often have a different injuring mechanism^39^ and are assessed through alternative clinical tests featured in previous diagnostic reviews.^59^ Although our meta-analysis excluded studies at a high risk of bias, the generalizability of our reported pooled diagnostic estimates to any specific setting might still be limited by reported differences in test technique, time since injury, reference standard used, and potential differences in referral time. Last, our proposed diagnostic algorithm of performing palpation and anterior drawer testing of the ATFL for accurate diagnosis has not yet been validated with patient paired data.

## Supplemental Material

sj-docx-1-sph-10.1177_19417381211029953 – Supplemental material for Diagnostic Accuracy of Clinical Tests Assessing Ligamentous Injury of the Talocrural and Subtalar Joints: A Systematic Review With Meta-Analysis Click here for additional data file. Supplemental material, sj-docx-1-sph-10.1177_19417381211029953 for Diagnostic Accuracy of Clinical Tests Assessing Ligamentous Injury of the Talocrural and Subtalar Joints: A Systematic Review With Meta-Analysis by Fredh Netterström-Wedin, Mark Matthews and Chris Bleakley in Sports Health: A Multidisciplinary Approach

sj-docx-2-sph-10.1177_19417381211029953 – Supplemental material for Diagnostic Accuracy of Clinical Tests Assessing Ligamentous Injury of the Talocrural and Subtalar Joints: A Systematic Review With Meta-Analysis Click here for additional data file. Supplemental material, sj-docx-2-sph-10.1177_19417381211029953 for Diagnostic Accuracy of Clinical Tests Assessing Ligamentous Injury of the Talocrural and Subtalar Joints: A Systematic Review With Meta-Analysis by Fredh Netterström-Wedin, Mark Matthews and Chris Bleakley in Sports Health: A Multidisciplinary Approach

sj-docx-3-sph-10.1177_19417381211029953 – Supplemental material for Diagnostic Accuracy of Clinical Tests Assessing Ligamentous Injury of the Talocrural and Subtalar Joints: A Systematic Review With Meta-Analysis Click here for additional data file. Supplemental material, sj-docx-3-sph-10.1177_19417381211029953 for Diagnostic Accuracy of Clinical Tests Assessing Ligamentous Injury of the Talocrural and Subtalar Joints: A Systematic Review With Meta-Analysis by Fredh Netterström-Wedin, Mark Matthews and Chris Bleakley in Sports Health: A Multidisciplinary Approach
