# Supplementary material for: Diagnostic Accuracy of Clinical Tests Assessing Ligamentous Injury of the Talocrural and Subtalar Joints: A Systematic Review With Meta-Analysis
Source: Sports Health. 2021 Jul 21;14(3):336–47. doi: 10.1177/19417381211029953 (PMC9109591; doi:10.1177/19417381211029953)
Supplement: sj-docx-1-sph-10.1177_19417381211029953 – Supplemental material for Diagnostic Accuracy of Clinical Tests Assessing Ligamentous Injury of the Talocrural and Subtalar Joints: A Systematic Review With Meta-Analysis [file sj-docx-1-sph-10.1177_19417381211029953.docx]

| **Appendix 1.** Contingency table with inconclusive test results grouped separately | | | |
| --- | --- | --- | --- |
| **Anterior drawer test** | Arthrography | | **Diagnostic yield** |
| **Prins 1978** | Injured (%) | Uninjured (%) |  |
| Positive | 67 (34) | 18 (18) | 29% |
| Negative | 63 (32) | 68 (67) | 44% |
| Total | 130 (66) | 86 (85) | **73%** |
| Intermediate results | 45 (23) | 11 (11) | 18% |
| Uninterpretable results | 22 (11) | 4 (4) | 9% |
| **Raatikainen et al 1982** |  |  |  |
| Positive | 69 (53) | 9 (16) | 41% |
| Negative | 33 (25) | 38 (67) | 38% |
| Total | 102 (78) | 47 (83) | **79%** |
| Uninterpretable results | 29 (22) | 10 (17) | 21% |
| **van den Hoogenband et al 1984** |  |  |  |
| Positive | 98 (65) | - | 65% |
| Negative | 13 (9) | - | 9% |
| Total | 111 (74) | - | **74%** |
| Intermediate results | 19 (13) | - | 13% |
| Uninterpretable results | 20 (13) | - | 13% |
| **van Dijk et al 1996** |  |  |  |
| Positive | 90 (77) | 9 (24) | 64% |
| Negative | 23 (20) | 25 (68) | 31% |
| Total | 113 (97) | 34 (92) | **95%** |
| Uninterpretable results | 4 (3) | 3 (8) | 5% |
| **Talar tilt test** |  |  |  |
| **Prins 1978** |  |  |  |
| Positive | 38 (19) | 4 (4) | 14% |
| Negative | 92 (47) | 84 (83) | 59% |
| Total | 130 (66) | 88 (87) | **73%** |
| Intermediate results | 16 (8) | 5 (5) | 7% |
| Uninterpretable results | 51 (26) | 8 (8) | 20% |
| **van den Hoogenband et al 1984** |  |  |  |
| Positive | 71 (47) | - | 47% |
| Negative | 32 (21) | - | 21% |
| Total | 90 (68) | - | **68%** |
| Intermediate results | 19 (13) | - | 13% |
| Uninterpretable results | 28 (19) | - | 19% |
