# Supplementary material for: Diagnostic Accuracy of Clinical Tests Assessing Ligamentous Injury of the Talocrural and Subtalar Joints: A Systematic Review With Meta-Analysis
Source: Sports Health. 2021 Jul 21;14(3):336–47. doi: 10.1177/19417381211029953 (PMC9109591; doi:10.1177/19417381211029953)
Supplement: sj-docx-2-sph-10.1177_19417381211029953 – Supplemental material for Diagnostic Accuracy of Clinical Tests Assessing Ligamentous Injury of the Talocrural and Subtalar Joints: A Systematic Review With Meta-Analysis [file sj-docx-2-sph-10.1177_19417381211029953.docx]

| **Appendix 2.** Summary table of study characteristics | | | | | |
| --- | --- | --- | --- | --- | --- |
| Author and year | Population characteristics | Study design | Index test(s) | Reference standard | Reference standard positive finding |
| Cho et al 2016 | N=28  9 F (32%)  Mean age 32.4±11.9) Chronic ankle instability | Retrospective review | Anterior drawer test | Arthroscopy | ATFL injury upon visualisation during arthroscopic assessment |
| Croy et al 2013 | N= 86  39 F (45%)  66 participants (mean age 22.7±3.6)  History of lateral ankle sprain  20 healthy controls (mean age 24.8±4.8) to determine cut-off for reference standard (control data not included in analysis) | Prospective study | Anterior drawer test | Stress ultrasound | ≥3.7mm talar translation during stress testing |
| De Simoni et al 1996 | N=30  15 F (50%)  Mean age 33 (19-65 min-max) Supination ankle injury | Prospective study | ATFL palpation  CFL palpation | MRI | 1: Partial rupture  2: Total rupture  3: Absent |
| Funder et al 1982 | N=444  Age 10-50 min-max  372 patients (159 F, 43%) with acute supination trauma underwent reference testing | Prospective study | Anterior drawer test  ATFL palpation  CFL palpation  Supination test  Heel adduction test  Talar tilt test | Arthrography | Leakage of contrast medium > 4cm^2^ laterally |
| George et al 2020 | N=35  18 F (51%)  Mean age 21.97±7.11  Ankle injury | Prospective study | Anterior drawer test  Talar tilt test | Stress ultrasound | 1: Partial rupture  2: Total rupture |
| Gomes et al 2017 | N=24  14 with symptoms  F 5(36%)  Mean age 28; 23-42 min-max  14 symptomatic, 5  History of lateral ankle sprain and CAIT score  10 controls (participant characteristics unknown) | Case-control | Anterior drawer test  Anterolateral talar palpation | MRI | Not defined |
| Gremeaux et al 2009 | N=34  14 F (41%)  27.4 mean age±4.2  acute ankle inversion injury, with a lateral ligament sprain diagnosis | Retrospective review | ATFL palpation  CFL palpation | Ultrasound | - Distension/edema/partial ATFL tear - Total ATFL and partial CFL tear - ATFL+CFL total tear |
| Li et al 2020 | N= 72 38 injured ankles from 31 participants [(13 F, 42%) (median age 30.4±8.9 years)] and 34 uninjured ankles from 29 participants [(14 F, 48%) (median age 29.1±8.9 years)]  History of one or more ankle sprains (more than one month since latest injury) | Prospective study | Anterior drawer test  Anterolateral drawer test  Reverse anterolateral drawer test | Ultrasound | ATFL injury based on the following criteria:   1. Partial or total ligament rupture 2. Lax ligament upon maximal plantar flexion/inversion 3. Thickened ligament, with a width of > 2.4mm or 20% above normal ligament width 4. No ligament fibres observed 5. Non-union of bony avulsions of the lateral malleolus |
| Lindstrand 1976 | 110 patients  30 F (27%)  mean age 26 years (14-47 min-max)  acute ankle injury with local pain | Prospective study | Anterior drawer test  Supination test  ATFL palpation  CFL palpation | Surgery | Fresh ATFL rupture, with or without concomitant injury to the CFL |
| Prins 1978 | 298 patients  90 F (30%)  mean age: 24.2 years  acute lateral ankle sprains | Prospective study | Anterior drawer test  Talar tilt test | Arthrography | 1: Anterolateral contrast medium leakage (ATFL rupture)  2: Anterolateral contrast medium leakage combined with leakage into the peroneal tendon sheath (ATFL+CFL rupture) |
| Raatikainen et al 1992 | 188 patients  44 F (23%)  Mean age 27 years (13-56, min-max)  acute inversion sprain with swelling, tenderness, bruises and/or difficulty walking. | Prospective study | Anterior drawer test | Arthrography | Anteroinferior leakage of contrast medium to the lateral malleolus (ATFL rupture) and/or leakage into the peroneal tendon sheath (CFL rupture) |
| van den Hoogenband et al 1984 | 150 patients  41 F (27%)  Mean age 27.6 (15-55, min-max)  Acute inversion sprain, with positive arthrogram and no history of previous severe ankle trauma | Clinical trial | Anterior drawer test  Talar tilt test | Arthrography | Leakage of contrast fluid:  1: ATFL rupture  2: ATFL+CFL rupture  3: ATFL+CFL+PTFL rupture |
| van der Ent 1984 | 732 patients  260 F (36%) with pressure pain, swelling, or skin discolouration | Prospective study | ATFL palpation  CFL palpation | Arthrography | Leakage of contrast fluid:  1: ATFL rupture  2: ATFL+CFL rupture  3: ATFL+CFL+PTFL rupture |
| van Dijk et al 1996 | 160 patients  44 (27.5%) female)  27.3 mean age (18–40 min-max)  Acute ankle injury | Prospective study | Anterior drawer test  ATFL palpation  CFL palpation | Arthrography | Lateral ligament lesion |
