# Supplementary material for: Diagnostic Accuracy of Clinical Tests Assessing Ligamentous Injury of the Talocrural and Subtalar Joints: A Systematic Review With Meta-Analysis
Source: Sports Health. 2021 Jul 21;14(3):336–47. doi: 10.1177/19417381211029953 (PMC9109591; doi:10.1177/19417381211029953)
Supplement: sj-docx-3-sph-10.1177_19417381211029953 – Supplemental material for Diagnostic Accuracy of Clinical Tests Assessing Ligamentous Injury of the Talocrural and Subtalar Joints: A Systematic Review With Meta-Analysis [file sj-docx-3-sph-10.1177_19417381211029953.docx]

| **Appendix 3.** Index test characteristics | | |
| --- | --- | --- |
| **Test**, author and year | Test protocol | Positive finding |
| **Anterior drawer test** |  |  |
| Cho et al 2016 | With the patient lying supine and the upper leg supported by a couch, an anterior linear force was applied to the talus | Partial to complete instability, with more severe laxity sometimes featuring the dimple sign |
| Croy et al 2013 | With the patient seated and the lower leg hanging off the edge of the bed with a flexed knee, the clinician stabilised the distal tibia with one hand and gently applied an anterior force to the calcaneus with the other hand. The foot was resting on the examiner's forearm, with the ankle in neutral (0 degrees) position. | Moderate to severe increases in laxity was considered a positive finding |
| Funder et al 1982 | With the patient seated and the ankle in 30 degrees of plantar flexion, the examiner stabilised the tibia whilst applying an anterior force to the calcaneus | Increased talar translation in comparison to contralateral ankle |
| George et al 2020 | With the patient seated and the lower leg hanging off the edge of the bed with a flexed knee, the clinician stabilised the distal tibia with one hand and gently applied an anterior force to the calcaneus with the other hand. The foot was resting on the examiner's forearm, with the ankle in 10-15 degrees of plantar flexion | Gross laxity with no endpoint |
| Gomes et al 2017 | With the patient seated or lying supine, the clinician stabilised the distal part of the leg with one hand and held the calcaneus with the other hand. The clinician then applied a posterior force to the tibia and an anterior force to the calcaneus, in a "coming and going" fashion. | Visual or hand proprioceptive instability observed by the examiner |
| Li et al 2020 | With the patient seated and the lower leg hanging off the edge of the bed with a flexed knee, the clinician stabilised the distal tibia with one hand and gently applied an anterior force to the calcaneus with the other hand. The foot was resting on the examiner's forearm, with the ankle in 10-15 degrees of plantar flexion | Moderate to severe increases in laxity was considered a positive finding |
| Prins 1978 | With the patient lying supine and the ankle in 30 to 60 degrees of plantar flexion, the calcaneus was pulled forward forcefully but gradually, whilst the other hand stabilised the tibia distally. | Talus felt moving from the mortise, accompanied with a sulcus sign unless excessively swollen |
| Raatikainen et al 1992 | The ankle was in neutral flexion and supination. | Increased joint laxity (instability) |
| van den Hoogenband et al 1984 | With the patient lying supine and the ankle in 30 to 60 degrees of plantar flexion, the calcaneus was pulled forward gradually, whilst the other hand stabilised the tibia distally. | Talus displaced from the mortise |
| van Dijk et al 1996 | With the patient seated or lying supine and the lower leg hanging off of the edge of the bed with a flexed knee, the clinician stabilised the distal tibia with one hand and gently applied an anterior force to the calcaneus with the other hand. The foot was resting on the examiner's forearm, with the ankle in 10-15 degrees of plantar flexion | Dimple sign or (sometimes) pain during stress testing |
| **Anterolateral drawer test** |  |  |
| Li et al 2020 | With the patient seated and the lower leg hanging off the edge of the bed with a flexed knee, the clinician stabilised the distal tibia with one hand and gently applied an anterior force to the calcaneus with the other hand's index and middle fingers. Simultaneously, the thumb was placed at the anterior aspect of the talar dome, 1cm proximal of the tip of the lateral malleolus, inspecting talus displacement. The ankle was kept in 10-15 degrees of plantar flexion throughout. | Moderate to severe increases in laxity was considered a positive finding |
| **Reverse anterolateral drawer test** |  |  |
| Li et al 2020 | With the patient lying supine and with a flexed knee, the foot was pressed firmly against the bed, maintaining 10-15 degrees of plantar flexion. The hand that pressed the foot against the bed had the index, middle finger and thumb positioned at the lateral ridge of the talar dome and its anterior aspect, 1cm proximal of the tip of the lateral malleolus, inspecting talus displacement. With the foot stabilised, the clinician applied a posterior force to the distal tibia with the other hand. | Moderate to severe increases in laxity was considered a positive finding |
| Lindstrand 1976 | Method 1: with the patient lying supine and the hip flexed about 70 degrees, and the knee flexed about 90 degrees and the ankle in slight plantarflexion (~10 degrees). One hand placed the foot in slight pronation and pressed firmly against the support, whilst the other hand applied a rapid and unexpected posterior force to the patient's tibia.  Method 2: If the patient struggled to relax during the method described above, the test was instead done with the patient lying supine with the entirety of their lower extremity resting against the bed. The examiner held the patient's heel in the cup of his hand and applied a posterior force to the tibia with the other hand. | Increased talar displacement in comparison to the contralateral ankle |
| **Anterolateral talar palpation** |  |  |
| Gomes et al 2017 | In the right ankle (kept at neutral), the examiner placed the left thumb in the area of the lateral talar ridge, allowing the examiner to feel for joint laxity during palpation. After the thumb was correctly placed, the remaining four fingers of the same hand grasped the lower leg. The right hand was then placed around the calcaneus to hold it still, whilst the left hand pushed down on the distal lower leg. The procedure was then repeated for the contralateral ankle. | Increased talar displacement in comparison to contralateral ankle |
| **ATFL palpation** |  |  |
| De Simoni et al 1996 | Not described | Direct ATFL tenderness |
| Funder et al 1982 | Not described | Direct ATFL tenderness |
| Gremeaux et al 2009 | Not described | Specific pain on palpation |
| Lindstrand 1976 | Not described | Tender region in the area of the ATFL |
| van der Ent 1984 | The palpating finger localised the tenderness punctum maximum along the lateral ligaments | ATFL pain on palpation |
| van Dijk et al 1996 | Not described | Specific pain on palpation |
|  |  |  |
| **CFL palpation** |  |  |
| De Simoni et al 1996 | Not described | Direct CFL tenderness |
| Funder et al 1982 | Not described | Direct CFL tenderness |
| Gremeaux et al 2009 | Not described | Specific pain on palpation |
| Lindstrand 1976 | Not described | Tender region in the area of the CFL |
| van der Ent 1984 | The palpating finger localised the tenderness punctum maximum along the lateral ligaments | CFL pain on palpation |
| van Dijk et al 1996 | Not described | Specific pain on palpation |
| **Supination test** |  |  |
| Funder et al 1982 | Internal rotation and supination of the foot | ATFL pain during stress testing |
| Lindstrand 1976 | Supination of the forefoot, with the other hand holding the lower leg well above the swollen and tender region | Ankle pain during stress testing |
| **Heel adduction** |  |  |
| Funder et al 1982 | Adduction of the heel | CFL pain during stress testing |
| **Talar tilt test** |  |  |
| Funder et al 1982 | Inversion of the calcaneus with a fixed tibia | Increased talar tilt in comparison to contralateral ankle |
| George et al 2020 | With the patient seated and the lower leg hanging off the edge of the bed with a flexed knee, the clinician stabilised the distal tibia with one hand and inverted the ankle with the other hand. | Gross laxity with no endpoint |
| Prins 1978 | In 30 to 60 degrees of plantar flexion, the examiner inverted the calcaneus forcibly, whilst the proximally placed hand gripped the lower leg around the tibiofibular mortise | The examiner's proximal hand feels the talus dislocating from the tibiofibular mortise during the manoeuvre |
| van den Hoogenband et al 1984 | With the patient seated and the ankle in 30 to 60 degrees of plantar flexion, the examiner inverted the calcaneus slowly but forcibly with one hand, whilst the other hand placed firmly around the calf | The examiner's proximal hand feels the talus subluxate from the tibiofibular mortise during the manoeuvre |
